# Medicaid Eligibility Gaps and Pandemic-Era Postpartum Insurance Rates

**DOI:** 10.1001/jamahealthforum.2025.0109

**Published:** 2025-03-21

**Authors:** Ellerie Weber, Heeun Kim, Annabelle Ng, Frances M. Howell, Ashley Fox, Teresa Janevic

**Affiliations:** 1 Department of Population Health Science and Policy, Icahn School of Medicine at Mount Sinai, New York, New York; 2 Department of Community Health Sciences, Fielding School of Public Health, University of California, Los Angeles; 3 Department of Epidemiology, Columbia University Mailman School of Public Health, New York, New York; 4 Department of Public Administration, Rockefeller College of Public Affairs and Policy, State University of New York at Albany, Albany, New York

## Abstract

This cohort study uses publicly available national survey data to estimate the association of the Families First Coronavirus Response Act with postpartum insurance rates.

## Introduction

The Families First Coronavirus Response Act (FFCRA), passed in response to the COVID-19 pandemic, effectively allowed eligible birthing people to remain continuously enrolled in Medicaid throughout the public health emergency, creating an unexpected opportunity to study the effects of long-advocated-for postpartum Medicaid extensions.^1^ This cohort study uses publicly available national survey data and a new study design to estimate the association of FFCRA with postpartum insurance rates.

## Methods

We used 2016-2022 American Community Survey microdata, omitting 2020 given known pandemic-related data collection issues.^2^ The sample comprised 196 936 (unweighted) postpartum people (22 311 310 weighted), defined as those 19 years and older reporting giving birth in the past 12 months. Outcomes were self-reported and included uninsurance, Medicaid, or private insurance.

The study design exploited pre-FFCRA variation in states’ Medicaid postpartum eligibility “gaps,” defined as the difference between states’ income eligibility thresholds for pregnant people vs nonpregnant parents.^3^ We used respondents’ reported income data to categorize them as exposed (ie, having incomes falling within their state’s gap, thus labeled “within gaps”) or controls. Controls had incomes either above pregnancy Medicaid threshold (above PM) or below parental Medicaid threshold (below TM) (eMethods in Supplement 1).

Using individual-level linear probability difference-in-differences models, we compared changes before and after FFCRA, in postpartum coverage among those exposed vs controls, adjusting for individual-level covariates (age, marital status, education, employment, race and ethnicity), time-varying state-level covariates (unemployment rate, poverty rate), and state fixed effects (eMethods in Supplement 1). We applied American Community Survey person weights and ran sensitivity analyses using an alternative gap measure and an above-PM cohort (eMethods in Supplement 1).

The institutional review board at Icahn School of Medicine at Mount Sinai approved this study. We followed STROBE reporting guidelines.

## Results

**Figure 1.**
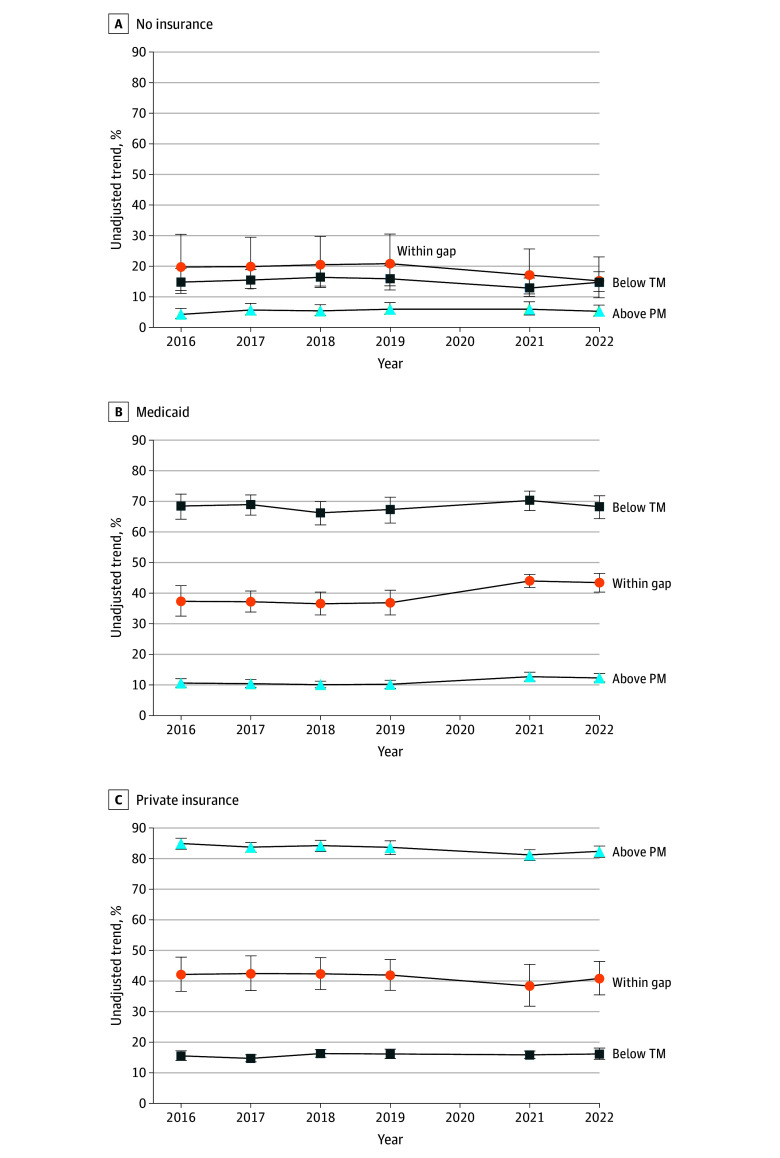
Unadjusted Trends in Uninsurance, Medicaid, and Private Insurance Rates by Exposure and Control Groups and Private Insurance Rates by Income Group Analysis of American Community Survey IPUMS data from 2016 to 2019 and 2021 to 2022. American Community Survey person-level weights are applied, and 2020 was excluded due to the COVID-19 pandemic. Below TM refers to the postpartum sample with incomes below their state’s income eligibility threshold for nonpregnant parents. Above PM refers to the postpartum sample with incomes above their state’s income eligibility threshold for pregnant people. Within gap refers to those with incomes between the TM and PM income eligibility thresholds, or, put differently, incomes between the eligibility thresholds for nonpregnant parents and those for pregnant people. Error bars indicate 95% CIs.

A total of 37 893 exposed postpartum people (5 404 519 weighted) and 158 746 controls (16 906 791 weighted) were identified. Unadjusted comparisons with controls show that in the year just before vs just after FFCRA exposed postpartum people reported more Medicaid coverage (36.8% vs 43.6%) and less uninsurance (20.2% vs 16.2%) (Figure 1).

**Figure 2.**
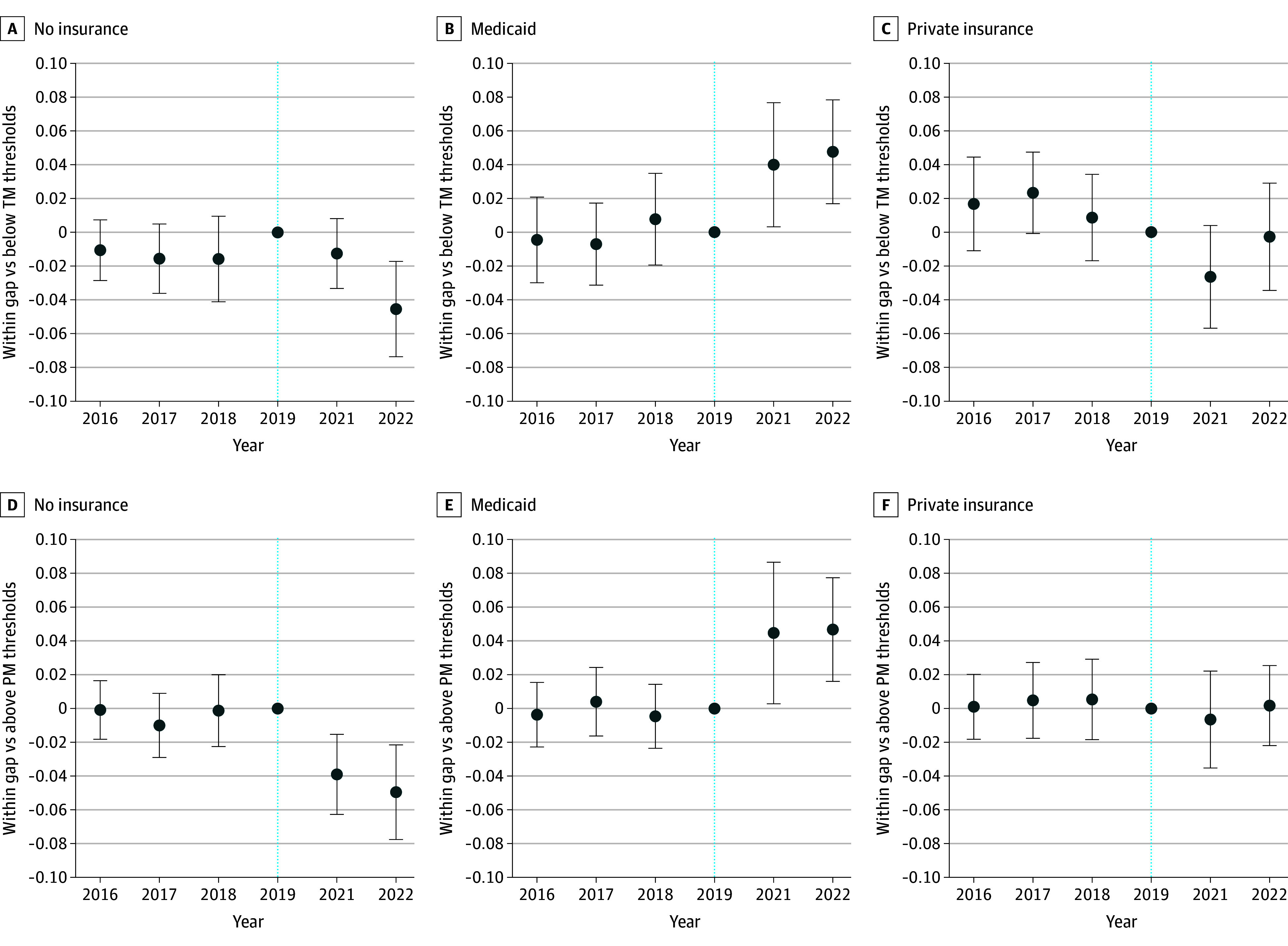
Adjusted Event Study Difference-in-Differences Coefficient Plots, 2016-2019 vs 2021-2022 Analysis of American Community Survey IPUMS data from 2016 to 2019 and 2021 to 2022, with adjusted coefficients from difference-in-differences regression. Results reflect American Community Survey person weights applied to individual-level variables. State fixed effects are included, and standard errors are clustered at state level. Below TM refers to the postpartum sample with incomes below their state’s income eligibility threshold for nonpregnant parents. Above PM refers to the postpartum sample with incomes above their state’s income eligibility threshold for pregnant people. Within gap refers to those with incomes between the TM and PM income eligibility thresholds, or, put differently, incomes between the eligibility thresholds for nonpregnant parents and those for pregnant people. The dotted lines at 2019 indicate it as the reference year before the Families First Coronavirus Response Act, and error bars indicate 95% CIs.

Adjusted regressions show that, relative to 2019, the likelihood of reporting Medicaid coverage increased by 4.0 percentage points (pp; 95% CI, 0.3-7.7 pp) in 2021 and 4.8 pp (95% CI, 1.7-7.9 pp) in 2022 for exposed postpartum people compared to below-TM controls (Figure 2). Their likelihood of reporting private insurance decreased by 2.6 pp (95% CI, −5.7 to 0.4 pp) in 2021 and 0.3 pp (95% CI, −3.4 to 2.9 pp) in 2022, relative to 2019. Together, the likelihood of uninsurance decreased for within-gap postpartum people compared to those below TM by 1.3 pp (95% CI, −3.3 to 0.8 pp) in 2021 and 4.8 pp (95% CI, −7.9 to −1.7 pp) in 2022, relative to 2019. Results were slightly stronger using the above-PM control group (Figure 2). Pre-FFCRA, there were no statistically significant differences in likelihood of exposure and control groups reporting Medicaid, private insurance, or uninsurance, confirming no pretrends.

## Discussion

Extending a burgeoning literature on FFCRA’s impact,^4,5^ we found that postpartum Medicaid extensions were most beneficial for individuals facing large eligibility gaps. Self-reported Medicaid coverage rates increased 4.0 to 4.8 pp among postpartum people within the gap—those most likely to lose Medicaid coverage pre-FFCRA—relative to controls likely unaffected by FFCRA. While private insurance decreased slightly more among the within-gap group, it did not offset Medicaid gains, so their uninsurance decreased 4.0 to 5.0 pp, relative to controls. This translates into 105 000 additional (weighted) within-gap postpartum people reporting Medicaid coverage who otherwise would not and 56 000 fewer reporting uninsurance.

Results imply that while uninsurance will be substantively lower among states with 12-month postpartum Medicaid extensions, gains in smaller-gap states may be limited. Other strategies to improve postpartum access to health care—such as raising income thresholds for parental Medicaid—should be considered.

Study limitations include a self-reported measure of insurance coverage that is unverifiable. Since FFCRA was implemented suddenly, postpartum people could have unknowingly retained Medicaid coverage.^6^ If true, these results might underestimate FFCRA’s impact.

## Notes

Supplement 1.
eMethods

Supplement 2.
Data Sharing Statement
