# Supplementary material for: Medicaid Eligibility Gaps and Pandemic-Era Postpartum Insurance Rates
Source: JAMA Health Forum. 2025 Mar 21;6(3):e250109. doi: 10.1001/jamahealthforum.2025.0109 (PMC11929033; doi:10.1001/jamahealthforum.2025.0109)
Supplement: Supplement 1. — eMethods [file jamahealthforum-e250109-s001.pdf]

Supplemental Online Content

Weber E, Kim H, Ng A, Howell FM, Fox A, Janevic T. Medicaid eligibility gaps and pandemic-era postpartum insurance rates. *JAMA Health Forum*. 2025;6(3):e250109. doi:10.1001/jamahealthforum.2025.0109

eMethods

This supplemental material has been provided by the authors to give readers additional information about their work.

© 2025 Weber E et al. *JAMA Health Forum*.

eMethods

Eligibility gap measure and exposure versus control classification

As mentioned, we defined our exposure group as those who fell in the Medicaid postpartum eligibility gap, defined as the difference between the state's income threshold for pregnant people ("pregnancy Medicaid" or "PM") and the state's threshold applicable to non-pregnant parents ("parental Medicaid" or "TM"). In contrast, we defined two control groups: (i) individuals "above-PM", and (ii) individuals "below-TM". Exposed postpartum people were most likely to benefit from continuous coverage, because they would have experienced a sharp eligibility cliff pre-FFCRA. Comparatively, control cohorts were likely unaffected by continuous coverage because, above-PM people would have never been eligible for Medicaid and below-TM group would have always been eligible for Medicaid.

To calculate whether respondents were in the control or treatment group, we used the IPUMS' "POVERTY" variable, which denotes an individual's adjusted household income relative to the official FPL, and compared it to the state's Medicaid income thresholds (per KFF). The "POVERTY" variable adjusts total household income by family size, the number of children, and the householder's age.

The larger the gap, the more exclusionary the state is to non-pregnant people, which means a greater risk of postpartum Medicaid beneficiaries losing coverage pre-FFCRA. While income thresholds are categorically higher for pregnant people than non-pregnant parents, the exact gap varies by state. For example, in 2019, Texas' income eligibility threshold was 207% of the FPL for qualifying pregnant people, and only 17% FPL for non-pregnant parents. Thus we calculated their gap as 190 percentage points. In another example, in 2019, Iowa's income eligibility threshold was 380% of the FPL for qualifying pregnant people, and 138% FPL for non-pregnant parents. Thus we calculated their gap as 242 percentage points. Distribution of the gap by state is available from the authors upon request.

Note, we used the highest threshold from any of possible pregnancy eligibility pathways available in a given person's state, because of the complexity and nuance of state Medicaid policy vis-à-vis the continuous coverage provision of FFCRA. Eligibility pathways include pregnancy Medicaid, CHIP Pregnant Woman, or the From-Conception-to-End-of-Pregnancy (FCEP) program (also known as "unborn child option" program). Not all states offer all programs, for example, only 17 states had a FCEP program in 2019. We did sensitivity analyses around this decision (described below).

© 2025 Weber E et al. *JAMA Health Forum*.

Event study difference-in-difference regressions

We specified the event DID model as follows:

$$Y_{it} = \alpha + \sum_{j=1}^{3} \beta_j pre_{ij} + \sum_{k=1}^{2} \beta_k post_{ik} + \mu X_{it} + \pi D_{st} + \varepsilon_{ist}$$

Subscript i indicates individual postpartum person, t indicates year, j is the number of pre-FFCRA years, and k is the number of post-FFCRA years. Our variable of interest is $post_{ik}$ a dummy variable that equals 0 for individuals in the control groups and 1 for individuals in the treated group in the post-FFCRA years (i.e., the interaction between between post- and the within-gap cohort dummies). In addition, X_{it} is a vector of individual-level covariates, and D_{st} is a vector of time-varying state characteristics. The DID coefficients β_k yield the change in probability of coverage in pre- vs post-FFCRA periods for the treatment group versus those in the control group. The event DID allows us to check formally for pre-trends by testing whether DID coefficients in pre-pandemic years are significantly different from zero.

We ran the regressions separately by control group, because we hypothesized they could experience different causal pathways. For example, compared to those in the below-TM cohort, the above-PM group may have greater access to private insurance and thus relative changes in private insurance rates may differ.

© 2025 Weber E et al. *JAMA Health Forum*.

Sensitivity Analysis: Alternative Gap Measure

As mentioned above, we used the highest threshold from any of possible pregnancy eligibility pathways available in a given person's state, because of the complexity and nuance of state Medicaid policy vis-à-vis the continuous coverage provision of FFCRA. Meaning, each state and indeed each program within each state can have additional eligibility criteria independent of the income threshold criteria. For example, typically the FCEP does not confer postpartum coverage, however some states still covered FCEP postpartum people during the public health emergency (PHE) period using state funds. Given the complexity of this, as well as the lack of information on which states covered which constituencies during the PHE, we wanted to use the most generous threshold to define the exposure (gap) measure.

We did sensitivity analyses excluding the FCEP threshold from the calculation and only use the highest of either the pregnancy Medicaid or CHIP Pregnant Woman programs. These were the two programs whose enrollees were guaranteed continuous coverage under FFCRA during the PHE, thus this is the most conservative gap measure.

Sensitivity Analysis: Upper Limit Income Cutoff (for the above-PM cohort)

As a second sensitivity analysis, we re-ran the event-study DID regressions considering an alternative above-PM control groups: those above the PM threshold but below 400% FPL. These are the people who "just missed" being eligible for Medicaid while pregnant, and thus are most likely to look similar on observables to the treated group.

Results from sensitivity analyses as well as unadjusted (descriptive) comparisons are available from the authors upon request.

© 2025 Weber E et al. *JAMA Health Forum*.
