# Supplementary material for: Medicaid Eligibility Gaps and Pandemic-Era Postpartum Insurance Rates
Source: JAMA Health Forum. 2025 Mar 21;6(3):e250109. doi: 10.1001/jamahealthforum.2025.0109 (PMC11929033; doi:10.1001/jamahealthforum.2025.0109)
Supplement: Supplement 2. — Data Sharing Statement [file jamahealthforum-e250109-s002.pdf]

Data Sharing Statement

Weber. Medicaid Eligibility Gaps and Pandemic-Era Postpartum Insurance Rates. *JAMA Health Forum*. Published March 21, 2025. doi:10.1001/jamahealthforum.2025.0109

Data
Data available: No

Additional Information
Explanation for why data not available: The ACS data used in this study are publicly available from the US Census Bureau. The authors can make the analysis code available upon request.
